# Factorial validity and invariance of the Patient Health Questionnaire (PHQ)-9 among clinical and non-clinical populations

**DOI:** 10.1371/journal.pone.0199235

**Published:** 2018-07-19

**Authors:** Satomi Doi, Masaya Ito, Yoshitake Takebayashi, Kumiko Muramatsu, Masaru Horikoshi

**Affiliations:** 1 Department of Global Health Promotion, Tokyo Medical and Dental University, Tokyo, Japan; 2 National Center of Neurology and Psychiatry, National Center for Cognitive Behavior Therapy and Research, Tokyo, Japan; 3 Graduate School of Clinical Psychology, Niigata Seiryo University, Niigata, Japan; North Carolina Neuropsychiatry Clinics, UNITED STATES

## Abstract

The Patient Health Questionnaire-9 (PHQ-9) is commonly used to screen for depressive disorder and for monitoring depressive symptoms. However, there are mixed findings regarding its factor structure (i.e., whether it has a unidimensional, two-dimensional, or bi-factor structure). Furthermore, its measurement invariance between non-clinical and clinical populations and that between patients with major depressive disorder (MDD) and MDD with comorbid anxiety disorder (AD) is unknown. Japanese adults with MDD (*n* = 406), MDD with AD (*n* = 636), and no psychiatric disorders (non-clinical population; *n* = 1,163) answered this questionnaire on the Internet. Confirmatory factor analyses showed that the bi-factor model had a better fit than the unidimensional and two-dimensional factor models did. The results of a multi-group confirmatory factor analysis indicated scalar invariance between the non-clinical and only MDD groups, and that between the only MDD and MDD with AD groups. In conclusion, the bi-factor model with two specific factors was supported among the non-clinical, only MDD, and MDD with AD groups. The scalar measurement invariance model was supported between the groups, which indicated the total or sub-scale scores were comparable between groups.

## Introduction

Depression is an exceedingly common comorbid condition in several mental disorders. To date, numerous self-report measures of depression have been developed, many of which are commonly used in clinical practice and research [1]. In particular, the Patient Health Questionnaire (PHQ) is one of the most useful measures for monitoring depressive symptoms and for screening for major depressive disorder (MDD) [2]. The 9-item version of the PHQ (PHQ-9) [3] is a brief and simple self-administered measure that corresponds with the nine diagnostic criteria for depressive disorder of the Diagnostic and Statistical Manual of Mental Disorders, Fourth Edition (DSM-IV). The PHQ-9 has been found to have high reliability and validity in Western populations, and can be used as a one- or two-item measure. The guidelines of the National Institute for Health and Clinical Excellence recommend the PHQ-9 for assessing the severity of depressive symptoms in clinical practice [4].

Despite its numerous advantages, the PHQ-9 has at least two main issues stemming from a lack of previous research, namely, the unclear factor structure and measurement invariance. First, there are mixed findings regarding the factor structure of the PHQ-9 in Western populations. Some studies using clinical samples (both in primary care and psychiatric settings) have determined that the PHQ-9 has a unidimensional factor structure [5]. In contrast, other studies on psychiatric patients or individuals with physical illness and depression have determined that a two-dimensional factor structure (including somatic and cognitive/affective symptom factors) had a better fit [6, 7, 8]. However, no studies have yet examined a possible bi-factor model that represents the existence of both a general factor and specific sub-factors called as group factors [9, 10]. For example, similar mixed results regarding the factorial unidimensionality of the Symptom Checklist-90-Revised has been resolved by testing a bi-factor model [11]. Hence, we hypothesized that the bi-factor model will show the best fit against the mere unidimensional or two-factor model.

Second, previous studies have not examined the possible measurement invariance [12] in the PHQ-9 between non-clinical and clinical populations. Additionally, no studies have yet examined whether the factor structure of the PHQ-9 could be assumed equivalently between patients with only MDD and those with MDD who comorbid anxiety disorder (AD). Given the high co-occurrence rate of depression and anxiety [13], it is necessary to examine the factor structure of the PHQ-9 between patients with only MDD and those with MDD who have comorbid AD.

In the present study, using an existing large dataset of non-clinical and clinical populations in Japan, we (1) compared the fit of the unidimensional, two-dimensional, and bi-factor models of the PHQ-9 via a confirmatory factor analysis; and (2) examined the measurement invariance between non-clinical, only MDD, and MDD with AD groups using a multi-group confirmatory factor analysis.

## Methods

### Participants and procedure

This study was part of a larger web-based survey for examining the emotions and psychopathology of Japanese clinical and non-clinical populations [14, 15]. We recruited participants in this study from panelists registered with Macromill Incorporation. This company is one of the largest Japanese internet marketing research company and has been used in previous studies [16]. A total of 2,830 individuals (1,547 females, 1,283 males; mean age = 42.44 years; *SD* = 10.39 years; range = 19−79 years) were selected randomly according to their age, gender, and living area from each population, including 619 individuals with MDD, 576 with social anxiety disorder, 619 with panic disorder, 645 with obsessive-compulsive disorder, and 371 without any psychiatric disorder (i.e., non-clinical population). The participants self-reported their own diagnoses by answering the following items regarding their current diagnoses and treatment of mental disorders: “Are you currently diagnosed as having Major Depressive Disorder and being treated for the problem in a medical setting?” Similarly, they were asked to respond “*yes*” or “*no*” to the question of their own diagnoses of social anxiety disorder, panic disorder, and obsessive-compulsive disorder. This study was approved by the institutional review board of the National Center of Neurology and Psychiatry (approval number: A2013-002).

### Measurements

#### Japanese version of the Patient Health Questionnaire-9 (J-PHQ-9)

The J-PHQ-9 assesses the frequency with which the nine symptoms of depression occurred over the last two weeks [17]. The participants rate each of the nine items on a scale ranging from 0 (*not at all*) to 3 (*nearly every day*). The reliability of the English version of PHQ-9 is excellent, as evidenced by the previous reports of an internal reliability Cronbach’s *α* of .86 to .91 [3, 5] and test-retest reliability [3]. The construct validity of the English version of the PHQ-9 confirmed by findings of previous studies which reported that increasing PHQ-9 scores were associated with worsening function [3, 18], increasing depression assessed using other measures [6, 18], increasing anxiety [6], and decreasing psychology well-being [6]. Additionally, in the present study, the internal reliability of the J-PHQ-9 was excellent, as evidenced by a Cronbach’s *α* .93, .84, and .91 for the total score, somatic score, and cognitive/affective score, respectively. The J-PHQ-9 also had good convergent validity as it was associated with the Japanese versions of the Kesseler Psychological Distress Scale (K6) [19] (*r* = .81) and Center for Epidemiologic Studies Depression Scale [20] (*r* = .86). In this study, we used the sum of the item scores of this scale for our analyses.

### Statistical analysis

**Fig 1:**
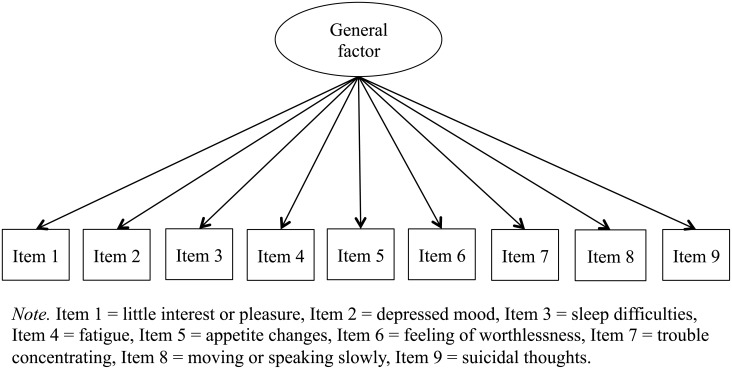
Unidimensional factor model.

**Fig 2:**
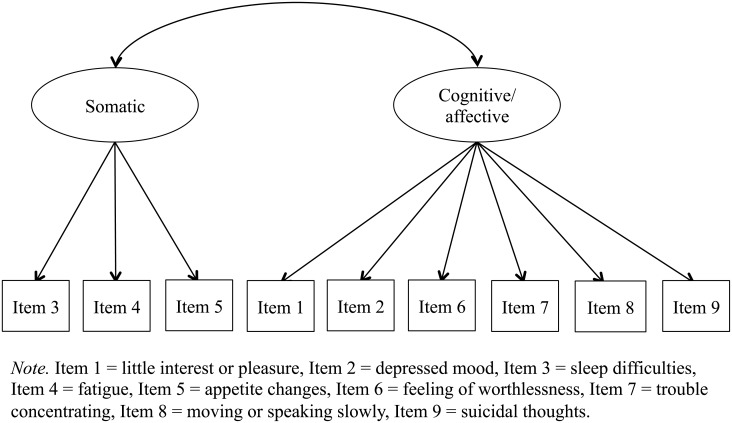
Two-dimensional factor model.

**Fig 3:**
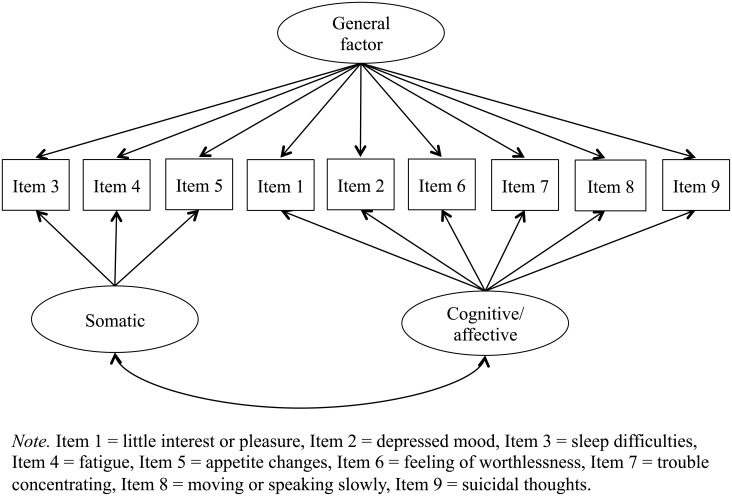
Bi-factor model.

First, we conducted a confirmatory factor analysis of the PHQ-9 using the data collected from the entire sample (*n* = 2,205). In this analysis, we determined and compared the fit of the above-stated three factor models to the data using the full information maximum likelihood method. In the unidimensional factor model, each item was represented by a single factor (Fig 1) [21]. In the two-dimensional factor model, items loaded onto one of the latent factors of somatic and cognitive/affective symptoms (Fig 2) [8]. Finally, in the bi-factor model, we designated somatic and cognitive/affective symptoms as specific group factors, and the sum of the item scores as the general factor (Fig 3).

Second, to examine the measurement invariance across non-clinical, only MDD, and MDD with AD populations, we conducted a multi-group confirmatory factor analysis [22]. We examined the measurement invariance of the PHQ-9 scores between the non-clinical and only MDD groups, and between the only MDD and MDD with AD groups. We constructed the following five increasingly restrictive models: where all parameters were free (Model 1: configural invariance); where loadings were invariant (Model 2: metric invariance); where loadings and intercepts were invariant (Model 3: scalar invariance); where loadings, intercepts, and residuals were invariant (Model 4: error variance invariance); and where loadings, intercepts, residuals, and factor means were invariant (Model 5: factor variance invariance). We used the following fit indices to evaluate the models: chi-square, root mean square error of approximation (RMSEA), Akaike information criterion (AIC), Bayesian information criterion (BIC), comparative fit index (CFI), and standardized root mean square residual (SRMR). Goodness-of-fit indices were examined in light of the following standards used in past literature [23]: the chi-square test (*χ*^2^) should not be significant; the RMSEA should be < .10 for acceptable fit and < .06 for good fit; the CFI should be ≥.90 for acceptable fit and >.95 for good fit; and the SRMR should be < .10 for acceptable fit and < .08 for good fit. The following criterion was used to adopting the model: a difference of less than .01 in the *Δ*CFI index supports the less parameterized model [24].

## Results

### Distribution of the PHQ-9 score

Mean values of the total, somatic, and cognitive/affective scores on the PHQ-9 were as follows: non-clinical group: 6.96 (Standard Deviation: SD = 6.46), 3.10 (SD = 2.62), and 3.86 (SD = 4.26); only MDD group: 12.42 (SD = 7.57), 5.16 (SD = 2.80), and 7.26 (SD = 5.27); MDD with AD group: 15.86 (SD = 7.20), 6.17 (SD = 2.58), and 9.69 (SD = 5.10).

### Confirmatory factor analysis

We compared the fit indices of the three models (unidimensional, two-dimensional, and bi-factor models) using the entire sample. The fit indices of the unidimensional model (*χ*^2^(27) = 1171.93, *p* < 0.001; RMSEA = .122; CFI = .936 SRMR = .037) and two-dimensional model (*χ*^2^(26) = 745.14, *p* < 0.001; RMSEA = .098; CFI = .960; SRMR = .029) were poorer than those of the bi-factor model (*χ*^2^(18) = 373.05, *p* < 0.001; RMSEA = .083; CFI = .980; SRMR = .020) (Table 1). Therefore, we selected the bi-factor model for subsequent analyses. In addition, Table 1 shows the fit indices of three models using the non-clinical, only MDD, and MDD with AD groups.

**Table 1:** The fit indices of the unidimensional, two-dimensional, and bi-factor models.

	*χ*^2^ value	df	RMSEA	CFI	SRMR
Entire sample					
Unidimensional model	1171.93	27	.122	.936	.037
Two-dimensional model	745.14	26	.098	.960	.029
Bi-factor model	373.05	17	.083	.980	.020
Non-clinical group					
Unidimensional model	354.40	27	.132	.914	.045
Two-dimensional model	196.88	26	.095	.958	.040
Bi-factor model	83.03	17	.074	.983	.031
Only MDD group					
Unidimensional model	223.03	27	.120	.920	.047
Two-dimensional model	179.54	26	.107	.938	.034
Bi-factor model	95.21	17	.094	.969	.021
MDD with AD group					
Unidimensional model	129.83	27	.105	.940	.041
Two-dimensional model	111.11	26	.097	.951	.037
Bi-factor model	36.32	17	.058	.989	.018

*Note*. df = degree of freedom, RMSEA = standardized root mean square residual, CFI = comparative fit index, and SRMR = standardized root mean square residual, MDD = Major depressive disorder, AD = Anxiety disorder.

Table 2 shows the standardized factor loadings for the bi-factor model using the entire sample. The general factor, the group cognitive/affective factor, and the group somatic factor accounted for 56.9%, 25.5%, and 17.6% of the common variance, respectively.

**Table 2:** Standardized factor loadings for the bi-factor model using entire sample.

	General	Cog/affect	Somatic
1. Little interest or pleasure	.579	.640	
2. Feeling down, depressed, or hopeless	.556	.737	
3. Trouble falling/staying asleep/sleeping too much	.497		.585
4. Feeling tired or having little energy	.530		.705
5. Poor appetite or overeating	.594		.479
6. Feeling bad about yourself/failure	.607	.531	
7. Trouble concentrating	.772	.294	
8. Moving or speaking so slowly	.749	.192	
9. Thoughts that you would be better off dead	.636	.439	
Factor correlation		.772	

*Note*. Cog/affect = Cognitive/affective.

### Multi-group confirmatory factor analysis

First, we conducted a multi-group confirmatory factor analysis (of the bi-factor model) for the non-clinical and only MDD groups (Table 3). According to the criterion for adopting the model, Model 3 showed the best fit (scalar invariance), wherein the loadings and intercepts were invariant.

**Table 3:** Summary of goodness of fit statistics for tested models in multi-group analyses.

	*χ*^2^	df	RMSEA	AIC	BIC	SRMR	CFI	*Δ*CFI
Non-clinical group vs. Only MDD group								
Model 1	176.55	34	.096	21724.96	22097.37	.025	.976	-
Model 2	225.81	52	.083	21738.21	22020.04	.073	.971	.005
Model 3	230.39	55	.081	21736.79	22003.52	.034	.971	0
Model 4	428.04	64	.102	21916.45	22137.88	.048	.939	.032
Model 5	520.86	67	.111	22003.26	22209.60	.106	.924	.015
Only MDD group vs. MDD with AD group								
Model 1	148.76	36	.089	17203.25	17203.26	.084	.976	-
Model 2	187.79	54	.079	17127.13	17127.13	.077	.969	.007
Model 3	210.92	60	.080	17105.43	17105.43	.076	.966	.003
Model 4	271.15	68	.087	17115.67	17115.67	.086	.950	.016
Model 5	452.50	71	.116	17276.77	17276.77	.116	.905	.045

Model 5 (Factor invariance model) = loadings, intercepts, residuals, and factor means are invariant.

*Note*. MDD = Major depressive disorder, AD = Anxiety disorder, RMSEA = Root Mean Square Error of Approximation, AIC = Akaike Information Criterion, BIC = Bayesian Information Criterion, CFI = Comparative Fit Index, SRMR = Standardized Root-Mean-Square Residual.

Model 1 (Configural model) = all parameter free.

Model 2 (Metric model) = loadings are invariant.

Model 3 (Scalar model) = loadings and intercepts are invariant.

Model 4 (Error invariance model) = loadings, intercepts, and residuals are invariant.

Second, we conducted a multi-group confirmatory factor analysis using only the MDD and MDD with AD groups (Table 3). Similar to the findings pertaining to the non-clinical and only MDD groups, Model 3 showed the best fit (scalar invariance). Scalar invariance indicates that differences in the factor mean lead to differences in item mean.

## Discussion

In this study, we compared a bi-factor model of the PHQ-9 with unidimensional and two-dimensional factor models and examined the measurement invariance of the PHQ-9 across non-clinical, only MDD, and MDD with AD groups. Among both non-clinical and clinical populations, we found that the bi-factor model had the best fit. This explains the mixed results found in previous studies that reported that the PHQ-9 has either a unidimensional or a two-dimensional factor structure [8, 21]. The bi-factor model allows one to use the unidimensional factor model of the PHQ-9, that is, we can use the cut-off point and the total score as a single variable. Additionally, we can use the two-dimensional factor model of the PHQ-9 for assessing more detailed symptoms. Moreover, the general PHQ-9 factor accounted for over 40% of the common variance. Thus, using both total score and sub-scale scores allows us to assess patients’ symptoms more precisely. In addition to assessing patients’ symptoms more precisely, we may be able to detect the change in patients’ symptoms due to treatment more fully by using the PHQ-9 regularly during treatment. This in turn will aid the implementation of appropriate treatment or the modification of the treatment according to the patients’ needs.

According to the results of the measurement invariance, scalar invariance showed best fit between the non-clinical and only MDD groups, and between the only MDD and MDD with AD groups, which means that we can compare the latent mean of the PHQ-9 between these two populations. Although the PHQ-9 total, somatic, and cognitive/affective scores of the MDD with AD group were higher than those of the MDD and non-clinical groups, these populations responded to each item similarly.

This study has several limitations. First, we might have obtained a biased sample because we conducted a web-based survey. For example, patients with more severe depressive symptoms and those who do not use the Internet frequently might have been excluded from this web-based survey. Second, participants were asked to report their own diagnoses and were not interviewed to assess whether they actually had MDD/AD. In other words, some of the participants might not have met the required diagnostic criteria for MDD/AD. This is in part supported by the low mean PHQ-9 score reported in the present study (*M* = 12.42 for MDD only, *M* = 15.86 for MDD/AD) as compared to that found in previous studies conducted in Western countries. For example, Kroenke [3] reported a mean score of 17.1 among 41 patients with MDD, and Petersen [8] reported a mean score of 17.3 among the 626 such patients. Future studies must test the higher-order factorial model and assess measurement invariance with participants diagnosed using a structured interview. Finally, we used only Japanese non-clinical and clinical populations, making it unclear whether these results are applicable to a Western population.

## Supporting information

S1 Dataset (XLSX)
